# A Historical Overview of Health Disparities and the Potential of eHealth Solutions

**DOI:** 10.2196/jmir.7.5.e50

**Published:** 2005-10-04

**Authors:** Michael C Gibbons

**Keywords:** access to information, socioeconomic factors, Internet, disparities, eHealth, minorities

## Abstract

Over the past decade, a rapidly expanding body of literature has demonstrated the existence of disparities in health and health care. While consensus has not emerged regarding the causes of disparities, they are generally thought to be related to sociocultural, behavioral, economic, environmental, biologic, or societal factors. To effectively address disparities, several authorities have suggested the need for greater information technology research and investments. eHealth researchers may be able to make significant contributions in this area through research and its applications. This paper begins with a historical overview of health disparities in the United States and Europe. It then discusses the role that the Internet, and access to the Internet, may play in the genesis of health disparities. Finally, this paper closes with a discussion of the potential benefits of eHealth applications and the possible contributions of the field to overcoming disparities in health and health care.

## International Origins

Over the past decade, a rapidly expanding body of scientific evidence has been put forth documenting differences in health status among US racial and ethnic groups. Evidence has also mounted suggesting that these differences may be related to both medical and nonmedical determinants. Internationally, however, neither the evidence nor the realization of a link between nonmedical sociobehavioral factors and health outcomes is new. The earliest reported observation of a hypothesized association between socioenvironmental risk factors and health outcomes occurred in Italy over three centuries ago when Bernardino Ramazzini detailed an unusually high frequency of breast cancer in Catholic nuns [1]. Not long thereafter, in 1775, British surgeon Sir Percival Pott reported a cluster of scrotal cancer cases among British chimney sweeps [1].

By the mid 19th century, large-scale epidemiologic evidence began to corroborate these early observations. In 1840, Edwin Chadwick, British civil servant and statistician, demonstrated mortality differentials between the social classes living in Liverpool, England. Chadwick asserted that these differences were likely due to poverty and lifestyle factors common to the poorer working classes [2]. German physician Rudolph Virchow went a step further when, in 1849, he asserted that, because diseases of the populace are traceable to defects in society, the focus of medicine should shift from changing the individual to that of changing the society [3]. Finally, in France, French physician Louis Villerme recommended improving school and working conditions as social interventions that would reduce class differences in mortality [3]. Thus, in Europe, by the beginning of the 20th century, the existence of class variations in morbidity and mortality were clearly evident in the scientific literature [2].

Throughout the 20th century, the study of social class differences in health status continued across Europe, especially in Britain were epidemiologists began using decennial census data to evaluate national mortality trends. The insights gained from these analyses enabled them to construct an occupational social class grading system that correlated inversely with infant mortality. It also was the basis of the claim made by the Registrar General of Britain that at least 40% of British infant mortality was entirely preventable if the social conditions of poor infants could be elevated to that of upper-class infants [2].

Two British researchers, Titmuss and Logan, evaluated regional class-based mortality trends and documented that the disparity in infant mortality rates between upper- and lower-class infants continued to increase from 1910 to 1950 [2]. This data, along with the Depression and World War II, encouraged the British government, in 1942, to respond by instituting the welfare state and promoting several policy initiatives designed to address the “five giants of Want, Disease, Ignorance, Squalor and Idleness” [2,4]. Despite this government investment, however, problems attributable to social inequalities and inadequate access to health care persisted. In fact, by the mid 1970s, some 30 years later, the evidence seemed to indicate that the problems were still increasing and that the health of British citizens was slipping behind that of other industrialized nations [4]. Thus, in 1977, the British government formed the Research Working Group on Inequalities in Health and selected Sir Douglas Black as its chair. The committee’s report, issued three years later in 1980, became known as the Black Report, and it represents the first attempt by a national government to systematically study, understand, and explain health inequalities [4]. In summary, the health improvement recommendations of the report emphasized the need to improve the physical and the social environment in which the poor and lower classes lived [4].

## Domestic Recognition

Across the Atlantic in the United States, scientific evidence from several lines of inquiry examining outcomes and patterns of health care delivered to defined populations began to converge and suggest the importance of the socioenvironment in determining health outcomes. Researchers using small area analysis and geographic information systems analytic techniques demonstrated that a significant amount of nonrandom practice variability existed between clinical practices in different geographic locales, despite treating clinically similar patients [5,6]. As public awareness grew, the US government became involved. In 1984, the US Department of Health and Human Services released a report on the health of the nation, entitled “Health, United States, 1983” [7]. The report documented that, while the overall health of the nation showed significant progress, major disparities existed in “the burden of death and illness experienced by blacks and other minority Americans as compared with the nation's population as a whole” [7].

In response to the disparities identified in the report, the secretary of the Department of Health and Human Services established a task force on black and minority health—the first time that the US government formed a group of experts to conduct a comprehensive study of minority health problems. In 1985, release of the “Report of the Secretary’s Task Force on Black and Minority Health” significantly raised awareness of the disparate health of the country’s minority groups as compared to the white majority population [8].

Large epidemiologic studies like the Harvard Medical Practice Study emerged, documenting that a significant portion of practice variability could be classified as substandard care and that there was a correlation between substandard care and health care centers treating substantial numbers of poor and minority patients [9-11].

The emerging problems of differential outcomes and health status were not limited however to minorities and the poor. The Whitehall studies of a large cohort of British civil servants had convincingly demonstrated that a social class–based health gradient existed even among the well educated and employed [12]. Additionally, it became increasingly recognized that certain community and societal level factors, including stress [13,14], early life experiences [15], social capital [16], and income inequality [17,18] seemed to exert significant effects on health and disease outcomes independent of personal behavior [3,19].

Soon, major philanthropic and advocacy organizations, including The Commonwealth Fund, The Kaiser Family Commission, the Kellogg Foundation, the Robert Wood Johnson Foundation, and The California Endowment began major initiatives designed to address issues related to disparities and health care quality [20].

By the late 1990s, the scientific evidence seemed to indicate that issues of disparity, practice variation, substandard care, and socioenvironmental determinants of health may all be related to the quality of health care experienced by patients. Friscella published his paper entitled “Inequality in Quality,” in which he called attention to issues of health care quality and health care disparities as related issues of health care organizational capacity. He further contended that national efforts to eliminate racial and ethnic disparities in health care and national health care quality improvement initiatives represented two inseparable components of providing high-quality health care for all citizens [21].

## Synthesizing the Scientific Evidence on Health Disparities

As the domestic evidence for population differences continued to accumulate, definitions of disparities were nonstandardized, and racial categorizations became increasingly criticized as being imprecise and biologically meaningless [22,23]. While multiple definitions are still in current use, disparities are generally held to be population differences in (1) environmental exposures, (2) health care access, utilization, or quality, (3) health status, or (4) health outcomes [24]. As alluded to above, within the US health care system these differences have most convincingly been demonstrated across racial and ethnic lines (whites vs minorities); however, disparities based on other categorizations have also been described, including geography (urban vs rural) [25], gender (male vs female) [26,27], socioeconomic status (poor vs nonpoor) [28,29], and age (nonelderly vs elderly) [30].

Health disparities are generally thought to be related to the health care system and other social factors. Several lines of investigation examining the socioenvironment and the clinical encounter give evidence of differences in the quality of care received by many racial and ethnic minorities. While these factors have been described as “causes” and are likely to be important in the genesis of disparities, scientifically validated evidence of definitive causal pathways and the underlying biologic mechanisms is largely lacking [31].

To help bring clarity to these issues, the Institute of Medicine (IOM) released the first of several reports highlighting and summarizing the scientific evidence concerning issues of differential health status, culture, behavior, communication, substandard care/medical errors, and health care quality [32-37]. The work of the IOM on disparity issues culminated with the 2003 release of a report entitled “Unequal Treatment: Confronting Racial and Ethnic Disparities in Health Care” [38]. In this report, the IOM Committee on Understanding and Eliminating Racial and Ethnic Disparities in Health Care was charged with assessing the extent and potential sources of racial and ethnic disparities in health care that are not otherwise attributable to access to care, ability to pay, or insurance coverage. The committee was also to provide recommendations regarding potential interventions to eliminate health care disparities [38]. The committee found that, within the United States, even among individuals with access to care, significant racial and ethnic disparities indeed existed and were related to historic and contemporary social and economic inequality, discrimination, and a fragmented US system of health care [38]. While the release of this report has engendered significant public, media, and academic interest, likely ensuring that efforts to understand and eliminate disparities will continue at least into the foreseeable future, the magnitude and intransigence of the problem, the complexity of its causal pathways, and its resistance to intervention efforts are only beginning to be realized [32].

## Digital Disparities

Since the mid-1990s when the World Wide Web became a powerful part of America’s communications and information culture, there has been great concern that the nation’s racial minorities would be further disadvantaged because Internet access was not spreading as quickly in the African-American community as it was in the white community. Former Assistant Secretary of Commerce Larry Irving said the following in his introduction to “Falling Through the Net,” the 1999 Department of Commerce Study on the digital divide (the divide between those with access to new information technologies and those without): “[The digital divide] is now one of America’s leading economic and civil rights issues” [39]. This report found that, although, overall, the number of Americans connected to the nation’s information infrastructure was soaring, a digital divide existed between whites and African-Americans in terms of their access to the Internet, and that, in many cases, the divide was *widening* over time. A follow-up study revealed a persistent but substantially narrowed gap, with large increases in computer ownership and Internet use across most major demographic populations [40]. The most recent survey, released in 2003, indicated a significant slowing in the growth of the number of Internet users since late 2001 [41]. Overall, 42% of surveyed individuals did not use the Internet, and significant utilization differences remained according to race, education, income, and geography (urban vs rural) [41]. Generally, whites are more connected than African-Americans and Hispanics. Even at equivalent levels of income, African-Americans are less likely to be online than whites or Hispanics. In fact, over the period of this study (mid-2000 to mid-2002), the composition of the non-Internet user group did not change substantially [41]. Interestingly, 56% of nonusers said they did not ever plan to go online and cited the cost of computers or Internet access, fear of fraud, credit card theft, or pornography as the major reasons for avoiding Internet use [41].

Recently, there has been a significant increase in the public availability of computers and Internet access at schools, public libraries, and workplaces [42]. Thus, conclusions regarding the extent of a digital disparity based on data considering only home-based access may be limited. Despite this reality, Internet availability in the home is accepted as an important indicator of equitable access among population groups [42]. In addition, access in public settings may be problematic because of computer monitoring in the workplace, privacy and confidentiality concerns, and the facilities’ hours of operation. Because of the potentially sensitive nature of health-related uses of the Internet, access at home is thought to be essential [42]. 

Several studies have shown that access to the Internet correlates with income level and educational attainment [39-42]. As with racial and ethnic differences, Internet utilization is increasing in all income brackets. The largest increases are seen in the higher income categories. All things considered, household incomes above US$50000 are positively associated with Internet utilization [41]. Beyond socioeconomic issues, some researchers have speculated that African-Americans have had less access to the Internet because they participate to a greater degree in entertainment-oriented technologies like television, rather than in information technologies. They argue that relatively high proportions of African-Americans use radio and television, but a relatively low proportion read newspapers [40]. As suggested above, the primary reasons why some groups have less access to information technology and resources are related to geography, literacy, disability, local infrastructure requirements, and cultural differences [43], some of which are not easily overcome simply by increasing personal computer ownership. Even if equity in personal computer and Internet access were achieved, emerging evidence suggests that online habits may vary by race and ethnicity. For example, online African-Americans are more likely than online whites to have (1) searched for information about major life issues such as researching new jobs and finding places to live, (2) used entertainment online, (3) used the Internet to obtain health information, and (4) searched for religious or spiritual information [40]. On the other hand, African-Americans with access to the Internet do not go online as often on a typical day as whites do, and they do not participate on a daily basis in most Web activities at the same level as online whites [40].

As information technology plays an ever-increasing role in Americans’ economic and social lives, the potential health implications of these findings need to be more clearly evaluated because the prospect that some people will be left behind in the information age may have serious repercussions [44]. Persistent digital disparities in access or utilization could leave some groups less able to take advantage of cutting edge innovations in population health technologies that enhance disease surveillance, environmental monitoring, food safety, emergency planning, disaster management, and geographic information systems–based tracking of environmental hazards [45].

## The Role of Information Technology in Overcoming Health Disparities

One major domain of eHealth focuses on improving health communication through the use of technology. This notion of enhancing communication and understanding is a fundamental component of addressing health disparities. Among other things, the recommendations of the Institute of Medicine report call for initiatives designed to enhance patient-provider communication, trust, and cultural appropriateness of delivered care [38]. Similar goals are the basis for the Healthy People 2010 objective to increase the number of individuals with Internet access in the home. Providers, health care organizations, and public health agencies are increasingly using the Internet as a main source of information dissemination and communication [42]. This need for innovative improvements in communication should represent a significant opportunity for eHealth technologies, researchers, and interventionists, with many important implications for overcoming disparities in health and health care. Given that eHealth is currently understood as attempting to facilitate the utilization of information technologies, the Internet, and communication technology in order to facilitate behavior change, improve health care, and enhance health outcomes [46], eHealth researchers may become the catalysts needed to spur the development of transdisciplinary interventions to effectively address disparities in health and health care.

Recent advances in the computer sciences and information technology fields have spawned several methodological advances in the biological and molecular sciences (eg, DNA chip technology and microarray analysis), enabled quantum leaps in molecular and submolecular medicine, and catalyzed the emergence of whole new fields of study such as proteomics, phenomics, nutrigenomics, and pharmacogenetics. Perhaps, in like manner, with the emergence of eHealth, the behavioral and population sciences may be on the verge of a similar information technology–based scientific revolution. New eHealth solutions may soon permit the real-time integrative utilization of vast amounts of behavioral-, biological-, and community-level information in ways not previously possible. Behavioral algorithms and decision support tools for scientists could facilitate the analysis and interpretation of population level data to enable the development of “community (population) arrays” or community-wide risk profiles, which in turn could form the foundation of a new “populomics.” This population-level risk characterization could potentially go beyond the limitations of typical geographic analyses and yield insights distinctly different from risk stratification based on current methodologies. Generically, these emerging technologies have been termed population health technologies and are believed to offer significant promise [45].

These assertions are not based on mere speculation. Encouraging early evidence suggests that multimedia health communication and behavior change efforts that include the use of computers and other eHealth technologies can improve health outcomes [47]. Among other factors, the evidence suggests that applications that are tailored to the individual, participatory, personally relevant, and contextually situated will be more likely to promote behavior change [47]. On the other hand, the Internet has been implicated in the causation or persistence of disparities because of the relative lack of access of some groups and because of its current inability to deliver content that is dynamically tailored to meet the cultural, language, or literacy needs of the individual user [48]. This may be particularly true of eHealth applications that are “Internet-enabled,” requiring access to the Internet to provide the interventional content. It is conceivably possible, however, to conceptually divide eHealth applications into at least two genres: those that rely on the Internet to deliver the interventional content directly to patients (Internet-enabled), and those that only employ the Internet to facilitate transfer and utilization of data for or about content that is delivered to patients by an alternate approach. The content or interventions themselves can actually function without the Internet, but when used in the context of the Internet, they are potentially much more efficacious and far-reaching. These types of technologies could be termed “Internet-enhanced” eHealth solutions. Here the Internet would facilitate the transfer of data and information, but the tailored content could be delivered by trusted people from the users own culture or community. The actual intervention could also be administered to patients by print or multimedia applications. Thus, in terms of overcoming health disparities, issues of guaranteeing Internet access for every individual may prove to be less important than attempting to address health disparities via interventions and methodologies that lack cultural relevance. Indeed, those interventions and strategies that integrate behavioral interventions with emerging information technologies will likely be the interventions capable of cost-effectively enabling mass customization, interactivity, and convenience. Ultimately, though, the health disparities challenge for eHealth researchers remains to harness the technical capabilities of emerging information technologies in ways that support the social and cultural realities in which people work and live [47], while enhancing our ability to address the health needs of every patient [49].
